# Unraveling the History and Revisiting the Synthesis of Degradable Polystyrene Analogues via Radical Ring-Opening Copolymerization with Cyclic Ketene Acetals

**DOI:** 10.3390/ma13102325

**Published:** 2020-05-19

**Authors:** Alexander W. Jackson, Srinivasa Reddy Mothe, Lohitha Rao Chennamaneni, Alexander van Herk, Praveen Thoniyot

**Affiliations:** Institute of Chemical and Engineering Sciences (ICES), 1 Pesek Road, Jurong Island, Singapore 627833, Singapore; alexander_jackson@ices.a-star.edu.sg (A.W.J.); srinivasa_reddy_mothe@ices.a-star.edu.sg (S.R.M.); lohitha_rao@ices.a-star.edu.sg (L.R.C.)

**Keywords:** cyclic ketene acetal, degradable, radical-ring opening polymerization, styrene

## Abstract

Degradable analogues of polystyrene are synthesized via radical ring-opening (co)polymerization (rROP) between styrene and two cyclic ketene acetals, namely 2-methylene-1,3-dioxepane (MDO) and 5,6-benzo-2-methylene-1,3-dioxepane (BMDO). This approach periodically inserts ester bonds throughout the main chain of polystyrene, imparting a degradation pathway via ester hydrolysis. We discuss the historical record of this approach, with careful attention paid to the conflicting findings previously reported. We have found a common ^1^H NMR characterization error, repeated throughout the existing body of work. This misinterpretation is responsible for the discrepancies within the cyclic ketene acetal (CKA)-based degradable polystyrene literature. These inconsistencies, for the first time, are now understood and resolved through optimization of the polymerization conditions, and detailed characterization of the degradable copolymers and their corresponding oligomers after hydrolytic degradation.

## 1. Introduction

The accumulation of man-made materials in the natural world is a topic of great concern. Currently, the chief culprit is plastic, specifically its increasing presence in our oceans [1]. Reducing, reusing and refurbishing plastic to the utmost extent possible will certainly play a crucial role in minimizing this problem. Nonetheless, there remains a need to develop polymer-based plastics which are easier to chemically breakdown before recycling, or transformation into other building blocks. The ultimate goal for polymer/plastic chemists working in this area is to increase the feasibility of circular economies. Free radical polymerization (FRP) is an extremely attractive approach when synthesizing polymers, as it is relatively cheap, experimentally straightforward and readily performed in aqueous media. FRP can provide access to a wide range of plastic-based materials due to the vast array of tried-and-tested vinyl monomers available. These benefits have resulted in FRP being employed to synthesize approximately 40–45% of all industrial polymers [2]. Perhaps the only drawback to chain-growth FRP is the resulting all carbon–carbon main-chain polymer, which furnishes polymer chains with incredible chemical stability. This chemical robustness is a double-edged sword, as it is responsible for both their highly desirable physical properties, and simultaneously their resistance to chemical breakdown and subsequent poor recyclability. With this in mind, one of the important tasks facing contemporary polymer chemists is to prepare degradable polymers via FRP. One of the essential objectives is to introduce chemically cleavable bonds within the main chain of FRP polymers which can facilitate their conversion into functional building blocks, after post-use collection. Of course, this incorporation of cleavable links should not significantly reduce the physical properties of the resultant material, a rather challenging balancing act.

**Figure 1:**
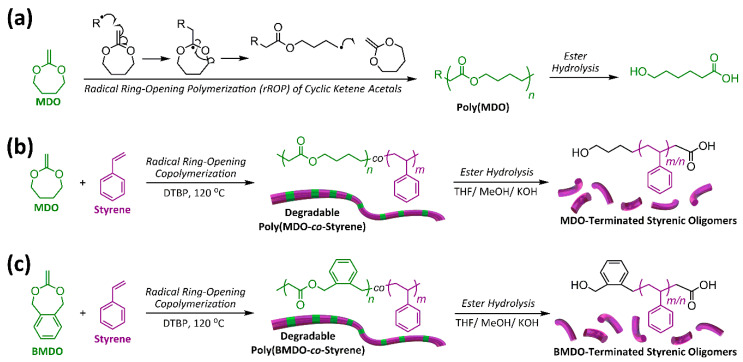
(**a**) Cyclic ketene acetal radical ring-opening polymerization (rROP) affording an aliphatic polyester; synthesis of degradable a polystyrene analogue via radical ring-opening copolymerization with (**b**) 2-methylene-1,3-dioxepane (MDO) affording poly(MDO-*co*-styrene) and (**c**) 5,6-benzo-2-methylene-1,3-dioxepane (BMDO) affording poly(BMDO-*co*-styrene).

In the early 1980s, the group of William J. Bailey published pioneering research describing the synthesis of linear aliphatic polyesters via radical ring-opening polymerization (rROP) of cyclic ketene acetals (CKAs) (Figure 1a) [3,4,5,6,7,8,9]. The most valuable aspect of this chemistry is the ability to copolymerize CKAs with conventional vinyl monomers, thereby periodically inserting hydrolytically cleavable ester bonds within the main chain of typically all carbon–carbon main-chain polymers. In doing so, a level of chemical degradability can be imparted into otherwise non-degradable vinyl polymers under FRP conditions. Since its discovery, CKA chemistry has been widely adopted to prepare degradable analogues of many conventional vinyl polymers [10,11]. Polystyrene is a common building block for a large range of plastic-based materials, including food packaging. Needless to say, the ability to synthesize a degradable polystyrene analogue would be a welcomed development in the effort towards tackling the build-up of plastic in the environment. Indeed, degradable analogues of polystyrene have been reported via copolymerization between styrene and CKA monomers. Interestingly, the literature reported thus far is full of conflicting outcomes, specifically around the level of CKA incorporation and subsequently the degree of polystyrene degradability. This retrospective article makes sense of the contradictory data previously published, by revisiting the rROP copolymerization between styrene and CKA monomers. We have found a ^1^H NMR characterization error, repeated throughout the existing body of work, that has resulted in a significant misinterpretation of the CKA-based degradable polystyrene results.

Essentially, the copolymerizations previously described in the literature are performed for extended periods of time with high levels of radical initiator, often resulting in a significant degree of monomer composition drift. Typically, styrene is primarily consumed first with a very low level of CKA incorporation, followed by the polymerization of the CKA monomer with a very low level of styrene incorporation. At first glance, the purified samples display high levels of ester incorporation, but the majority of polystyrene contains a very low degree of CKA units. In order to truly confirm the degree of CKA incorporation and degradability, we have found that it is vital to link the purified polymer compositions to the degraded oligomer molecular weight post-hydrolysis. Herein, we outline a conclusive copolymerization study with careful attention paid to ^1^H NMR characterization of the copolymers and the oligomers obtained after poly(CKA-*co*-styrene) degradation. First, we walk through the interesting history of degradable polystyrene analogues via rROP with CKAs.

The journey begins in 1982, with the Bailey group reporting an equimolar copolymerization of the CKA monomer 2-methylene-1,3-dioxepane (MDO) and styrene furnishing degradable poly(MDO-*co*-styrene) containing 23.4 mol % of MDO (Figure 1b) [3]. Similar results were published the same year which describe another equimolar copolymerization—this time between styrene and 5,6-benzo-2-methylene-1,3-dioxepane (BMDO) affording degradable poly(BMDO-*co*-styrene) (Figure 1c), with a BMDO incorporation of 31.1 mol % [4]. Finally, another publication from 1982 described a third equimolar copolymerization, between styrene and 2-methylene-4-phenyl-1,3-dioxolane (MPDL) affording degradable poly(MPDL-*co*-styrene) with a MPDL incorporation of 31.7 mol % [5]. These early findings suggest relatively agreeable copolymerizations and point towards similar monomer reactivities between styrene and various CKA monomers. These ground-breaking polymerizations published by the Bailey group were often performed for 24–36 h, in bulk, at 120 °C with between 1 and 3 mol % di-*tert*-butyl peroxide (DTBP) as the radical source—conditions that we believe can result in significant composition drift. Contrary to these original reports, they go on to describe a copolymerization with an 80: 20 molar feed ratio of MDO: styrene which resulted in a polymer containing only 10 mol % of MDO [7], and these results point towards very disparate monomer reactivity ratios. They continued their work and in 1990 published similar results—this time, under semi-batch conditions, using reactivity ratios of *r*_styrene_ = 23.6 and *r*_MDO_ = 0.021 [9]. They reported levels of MDO incorporation between 5 and 18 mol %, which resulted in between 0.26% and 2.76% biodegradability after 102 days. The innovative work performed by the Bailey group is undeniably revolutionary and has inspired many research groups since their initial discovery. However, by modern standards, their work lacks detailed polymer characterization, especially with respect to the degraded styrenic oligomers.

The story was picked up in 1993 by Hiraguri and Tokiwa, who describe a rROP between styrene and the CKA monomer 2-methylene-1,3,6-trioxocane (MTC) [12]. They performed an equimolar copolymerization and claimed a 24 mol % incorporation of MTC. However, at 120 °C for 24 h with 3 mol % DTBP radical initiator, they likely encountered severe composition drift, and while they probably have 24 mol % CKA in the purified sample, this is not necessarily an accurate degree of incorporation throughout the polystyrene prepared. They provide no molecular weight analysis of the styrenic oligomers obtained after ester hydrolysis.

**Figure 2:**
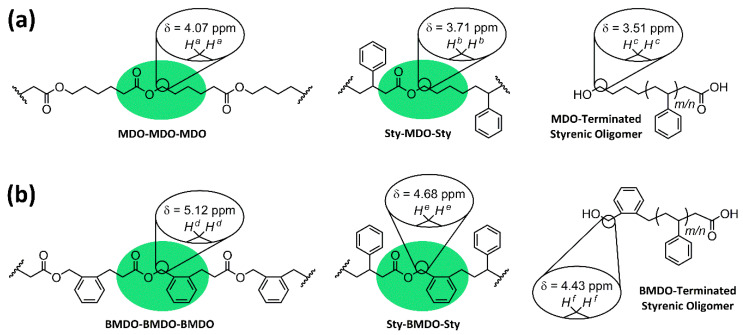
Illustration of key protons utilized for ^1^H NMR (CDCl_3_) characterization of poly(CKA-*co*-styrene) copolymers and degraded styrenic oligomers within (**a**) poly(MDO-*co*-styrene) copolymers and (**b**) poly(BMDO-*co*-styrene) copolymers.

In 2001, the Davis group attempted a pulse-initiated copolymerization between styrene and MDO [13]. They conclude that there is no incorporation of MDO due to the lack of a peak at δ = 4.07 ppm (believed to correspond to the methylene protons present in the ring-opened MDO ester C(O)OC***H_2_***H_2_). This characterization method was previously used by Bailey. However, we believe the presence of a peak at δ = 4.07 ppm (*H^a^*) relates to polymers very rich in MDO, essentially homo-polyMDO formed towards the end of the polymerization, and is a result of a MDO-centered triad with MDO on both sides (Figure 2a). We have found that the MDO ester protons (C(O)OC***H_2_***H_2_) appear at δ = 3.71 ppm (*H^b^*) for the MDO-centered triad in between two styrene units (Figure 2a), discussed in detail later. A very close look at the ^1^H NMR spectra presented by Davis (Figure 2 in [13]) does appear to display a small peak at δ ≈ 3.7 ppm. Frustratingly, the image is of rather low resolution and it is hard to be conclusive, but it could be the case that they did after all incorporate a small degree of MDO. 

The rROP between styrene and the CKA monomer 4,7-dimethyl-2-methylene-1,3-dioxepane (DMMDO) has also been performed under *‘living’* radical polymerization conditions, namely atom transfer radical polymerization (ATRP). In this report, an equimolar copolymerization resulted in poly(DMMDO-*co*-styrene) with a 4.6 mol % DMMDO incorporation [14]. ATRP has also been employed to mediate the copolymerization between styrene and BMDO with an equimolar copolymerization affording poly(BMDO-*co*-styrene) with a 19.0 mol % BMDO incorporation [15]. It is important to note that under ‘*living*’ polymerization conditions, polymer chains continue to grow throughout the experiment and, therefore, a significant monomer composition drift would furnish ‘*blocky*’ gradient copolymers rather than two distinct chemical composition distributions. In the second example, the authors note that poly(BMDO-*co*-styrene) prepared via ATRP possessed two glass transition temperatures (*T*_g_), likely due to immiscible blocks as a result of the blocky nature of the copolymer. The variation in CKA incorporation could be due to DMMDO and BMDO possessing different reactivities and propagating radical stabilities. However, in the case of poly(DMMDO-*co*-styrene), the polymerization was performed for 24 h, compared to 72 h for poly(BMDO-*co*-styrene)—this extended reaction time, significantly beyond near complete styrene conversion, is likely the cause of increased CKA content.

The most recent development was reported in 2007 [16]—MDO and styrene were copolymerized for 12–36 h with 2 mol % DTBP radical initiator, and a large peak is visible in the ^1^H NMR spectra presented (Figure 2 in [16]), which is identified as the MDO content within poly(MDO-*co*-styrene). However, under the conditions described, monomer composition drift is highly likely, and this peak could correspond to polymer chains very rich in MDO, formed after most of the styrene is consumed. The spectra might also display a peak at δ ≈ 3.7, which corresponds to the MDO content present in the polystyrene chains. Again, this spectrum is of very low resolution and it is hard to see the key peak. In this work, they claim a series of polymers with MDO levels of 6.5–21.7 mol %. Degradation is performed on poly(MDO-*co*-styrene) with 21.7 mol % MDO, but this does not result in any oligomers below a number average molecular weight (*M*_n_) of 4000 Da. An MDO incorporation of 21.7 mol % should furnish degraded oligomers with a *M*_n_ of 490 Da. This would suggest that their reported CKA content is accurate for the purified polymer sample, but not representative of the majority of polystyrene chains.

This retrospective article probes the copolymerization of styrene with either MDO or BMDO in an attempt to achieve full consistency between the NMR spectra of the original copolymer (with the corrected peak assignments) and the degraded styrenic oligomer molecular weights. Variations in reaction time, the initiator concentration and monomer feed compositions are investigated. The copolymer CKA incorporation is compared to the corresponding degraded styrenic oligomer molecular weight in order to determine the feasibility of CKA incorporation.

Throughout this manuscript, the theoretical number average molecular weight (*M*_n_^theo^) of the degraded oligomers is calculated using the equation below:

$$\mathrm{Degraded}\;\mathrm{Oligomer}\;{M_{n}}^{\mathrm{theo}}=\left(\left(\frac{\mathrm{styrene}\;\mathrm{molar}\;\mathrm{composition}}{\mathrm{CKA}\;\mathrm{molar}\;\mathrm{composition}}\right)\;\times \;\mathrm{styrene}\;m_{r}\right)+\mathrm{CKA}\;m_{r}$$

styrene *m*_r_ = molecular weight of one styrene repeat unit (104.15 Da), and CKA *m*_r_ = molecular weight of one CKA repeat unit (MDO = 114.14 Da and BMDO = 162.19 Da).

## 2. Materials and Methods

### 2.1. Chemicals

All chemicals were purchased from Sigma Aldrich (Saint Louis, MO, USA) and used as received, except styrene, which was passed through a basic alumina column prior to use. The 2-methylene-1,3-dioxepane (MDO) [3] and 5,6-benzo-2-methylene-1,3-dioxepane (BMDO) [4] were prepared as previously reported. 

### 2.2. Analytical Techniques

The ^1^H NMR spectra were recorded on a Bruker 400 Ultra Shield spectrometer (Billerica, MA, USA). Size exclusion chromatography (SEC) was conducted on a Viscotek TDAmax (Malvern Instruments, Worcestershire, UK) consisting of a GPCmax integrated solvent sample delivery module, a TDA 302 Triple Detector Array (Malvern Instruments, Worcestershire, UK), and OmniSEC software (Version 10, Malvern Panalytical, Egham, UK). Further, 2 × PLgel 5 µm Mixed-C (200–2,000,000) columns were applied for separation. THF was used as the eluent at 1.0 mL/min and 30 °C, ad molecular weights were determined against polystyrene standards.

### 2.3. Synthesis of Polystyrene (P1)

Styrene (5.21 g, 50 mmol) and di-*tert* butyl peroxide (73 mg, 0.5 mmol, 1 mol %) were transferred into a 25 mL Schlenk tube. The reaction mixture was degassed via three freeze–pump–thaw cycles and backfilled with N_2_. The reaction was heated to 120 °C and stirred at 500 rpm for 36 h. After this time, ^1^H NMR spectroscopy (CDCl_3_) was used to determine a conversion of 99%. The reaction was quenched by rapid cooling, and the polymer purified by three precipitations from CH_2_Cl_2_ into methanol. The purified polymer was isolated as a white powder (4.64 g, 89% yield).

### 2.4. Synthesis of PolyMDO (P5)

A 25 mL Schlenk tube was rinsed with Et_3_N and dried under high vacuum. The 2-methylene-1,3-dioxepane (5.71 g, 50 mmol) and di-*tert* butyl peroxide (73 mg, 0.5 mmol, 1 mol %) were transferred into the Schlenk tube. The reaction mixture was degassed via three freeze–pump–thaw cycles and backfilled with N_2_. The reaction was heated to 120 °C and stirred at 500 rpm for 36 h. After this time, ^1^H NMR spectroscopy (CDCl_3_ passed over Na_2_CO_3_) was used to determine a conversion of 78%. The reaction was quenched by rapid cooling, and the polymer purified by three precipitations from CH_2_Cl_2_ into hexane. The purified polymer was isolated as a clear waxy solid (4.09 g, 92% yield).

### 2.5. Synthesis of Poly(MDO-co-styrene) (P2–P4 and P6–P9)

Variations in monomer ratio, the initiator concentration and reaction time are outlined in Table 1. A typical polymerization (**P4**) is performed as follows. A 25 mL Schlenk tube was rinsed with Et_3_N and dried under high vacuum. The 2-methylene-1,3-dioxepane (2.85 g, 25 mmol), styrene (2.60 g, 25 mmol) and di-*tert* butyl peroxide (73 mg, 0.5 mmol, 1 mol %) were transferred into the Schlenk tube. The reaction mixture was degassed via three freeze–pump–thaw cycles and backfilled with N_2_. The reaction was heated to 120 °C and stirred at 500 rpm for 36 h. After this time, ^1^H NMR spectroscopy (CDCl_3_ passed over Na_2_CO_3_) was used to determine monomer conversions (99% styrene and 30% MDO). The reaction was quenched by rapid cooling, and the polymer purified by three precipitations from CH_2_Cl_2_ into methanol to remove unreacted MDO. The purified polymer was isolated as a white powder (3.27 g, 94% yield).

### 2.6. Synthesis of Poly(BMDO-co-styrene) (P10–P12)

Variations in the initiator concentration and reaction time are outlined in Table 1. A typical polymerization (**P10**) is performed as follows. A 25 mL Schlenk tube was rinsed with Et_3_N and dried under high vacuum. The 5,6-benzo-2-methylene-1,3-dioxepane (4.05 g, 25 mmol), styrene (2.60 g, 25 mmol) and di-*tert* butyl peroxide (73 mg, 0.5 mmol, 1 mol %) were transferred into the Schlenk tube. The reaction mixture was degassed via three freeze–pump–thaw cycles and backfilled with N_2_. The reaction was heated to 120 °C and stirred at 500 rpm for 36 h. After this time, ^1^H NMR spectroscopy (CDCl_3_ passed over Na_2_CO_3_) was used to determine monomer conversions (99% styrene and 22% BMDO). The reaction was quenched by rapid cooling, and the polymer purified by three precipitations from CH_2_Cl_2_ into methanol. The purified polymer was isolated as a white powder (3.14 g, 90% yield).

### 2.7. Determination of Monomer Conversions and Polymerization Yield

Styrene conversion was determined by comparing the residual vinyl peaks (δ 5.16 and 5.71 ppm) with the entirety of the aromatic region (δ 6.25–7.25 ppm) which contains polymerization and residual styrene units. MDO conversion was determined by comparing residual MDO (δ = 3.40 ppm) against polymerized MDO units (δ = 3.70–4.15 ppm). BMDO conversion was determined by comparing residual BMDO (δ = 3.72 ppm) against polymerized BMDO units (δ = 4.60–5.20 ppm). Polymerization yield = (mass of polymer obtained/(monomer conversion × original monomer mass)) × 100.

### 2.8. Degradation of Copolymers

The degradation process for each copolymer (**P2–P4**, **P6–P9** and **P10–P12**) is as follows. The copolymer (100 mg) is transferred into a 15 mL vial fitted with a magnetic stirrer. THF (4 mL) is added and the polymer is allowed to dissolve. To this solution, KOH (200 mg in 800 mg MeOH) is added. The addition of 20% methanolic KOH sometimes results in minimal polymer precipitation. To ensure a homogeneous degradation reaction, THF is added dropwise until complete polymer dissolution is observed. The degradation is carried out for 48 h at room temperature while stirring at 500 rpm. After this time, 0.5 mL conc. HCl(aq) is added, followed by evaporated to dryness. CHCl_3_ (5 mL) is then added and the heterogeneous solution obtained is stirred for 2 h at room temperature, and the KCl precipitate is removed by filtration and the degraded oligomer obtained after evaporation of the filtrate. The degraded oligomers are analyzed directly by GPC in order to ensure no oligomer fractionation. The ^1^H NMR spectra are obtained after one precipitation from CH_2_Cl_2_ into methanol, and precipitations proceeded with 80–90% oligomer mass recovery.

## 3. Results and Discussion

**Figure 3:**
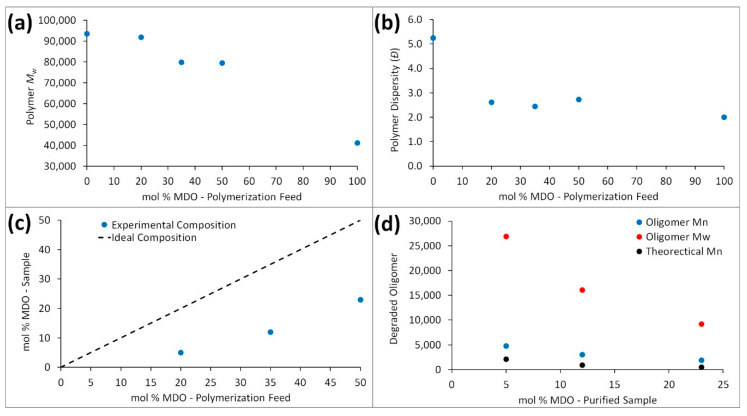
Polymer analysis for **P1–P5** displaying mol % of MDO in polymerization feed against (**a**) polymer molecular weight (*M*_w_) and (**b**) polymer dispersity (*Đ*). Polymer analysis for **P2–P4** displaying (**c**) mol % of MDO in polymerization feed against mol % MDO in a purified sample and (**d**) mol % MDO in purified samples against degraded oligomer molecular weight.

The primary aim of this work is to unravel the contradictory results previously reported in the literature. The conditions and characterization of each polymerization are presented in Table 1. Initially, we performed a series copolymerizations (**P2–P4**) between MDO and styrene under the conditions reported by the Bailey group in 1982 (120 °C, 36 h, 1 mol % DTBP, in bulk), including a homopolymerization of styrene (**P1**) and MDO (**P5**). A decrease in polymer molecular weight was observed with increasing MDO presence in the polymerization feed (Figure 3a), suggesting either increased levels of termination or the presence of chain-transfer reactions with increasing MDO content. The dispersity (*Đ* = 5.25) obtained for polystyrene (**P1**) was significantly higher than the poly(MDO-*co*-styrene) copolymers (**P2–P4**) and polyMDO (**P5**) (Figure 3b), and this could be a result of lower overall monomer conversion and/or suggest a degree of transfer to monomer. The equimolar copolymerization (**P4**) afforded a purified sample with an MDO composition of 23 mol %, nearly identical to the composition reported (23.4 mol %) by Bailey in 1982 [3]. This suggested that lowering the initial MDO feed composition would still result in some degree of MDO in the purified sample, and this was indeed observed for **P3** (35 mol % feed: 12 mol % sample) and **P2** (20 mol % feed: 5 mol % sample). Even though the MDO level present in the purified sample differed from the initial feed composition (Figure 3c), the presence of signals corresponding to the protons of the MDO repeating units of MDO homopolyester are clearly visible in the ^1^H NMR spectra of **P2–P4**. The key question at this stage is whether these MDO monomer units represent almost pure MDO homopolymer or significant incorporation within the polystyrene chains. Crucially, the molecular weight of each copolymer’s corresponding oligomeric degradation product varied significantly from the theoretical values calculated (Figure 3d), and we observed very large oligomer dispersities (*Đ* = 4.84–5.60) indicating a broad chemical composition distribution within the original sample. These initial findings implied the possibly of substantial monomer composition drift. As every copolymer (**P2–P4**) successfully underwent degradation, it is clear that the styrene polymerization does proceed with MDO incorporation, whereas the degraded oligomer molecular weights and dispersities suggest that a large portion of MDO is consumed towards the end of the reaction, resulting in polymer chains comprised almost entirely of MDO.

**Figure 4:**
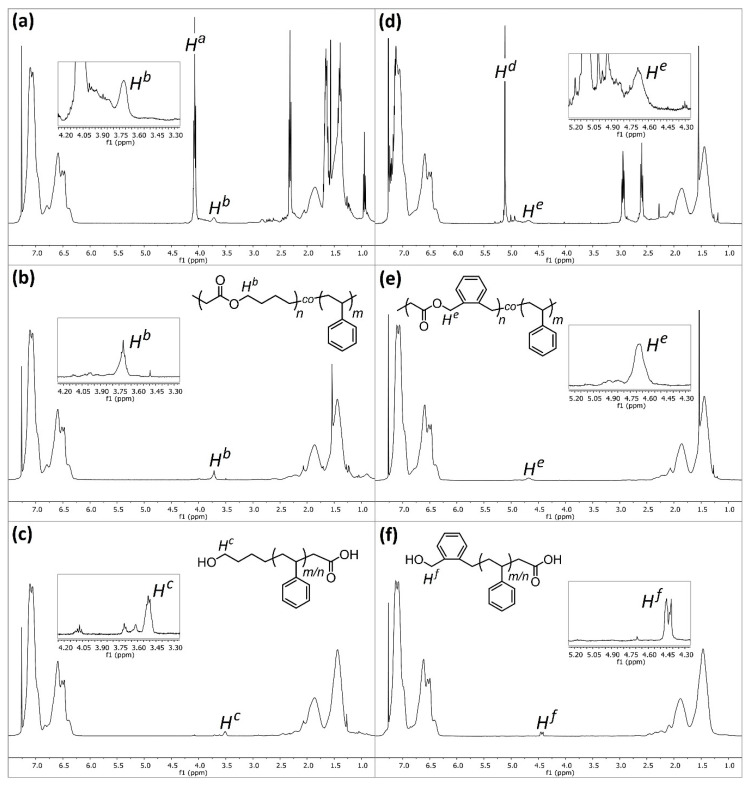
The ^1^H NMR (CDCl_3_) spectra of (**a**) **P4**, (**b**) **P6**, (**c**) degraded oligomers of **P6**, (**d**) **P10**, (**e**) **P11** and (**f**) degraded oligomers of **P11**.

**Figure 5:**
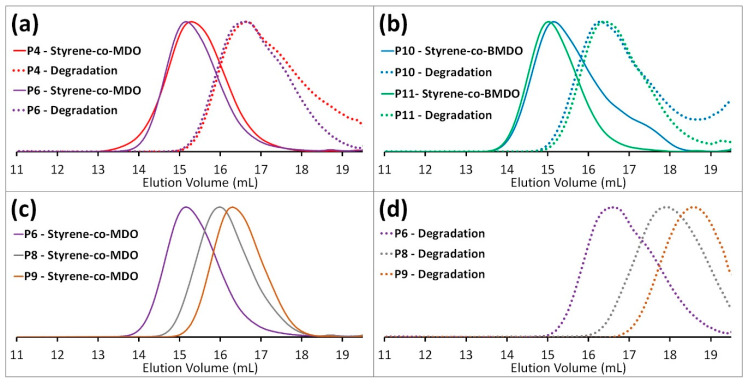
SEC traces of selected degradable poly(CKA-*co*-styrene) copolymers and of their corresponding oligomeric degradation products obtained via ester bond cleavage, (**a**) polymers **P4**-**P6** original and degradation SEC traces, (**b**) polymers **P10** and **P11** original and degradation SEC traces, (**c**) **P6**, **P8** and **P9** original polymer SEC traces, (**d**) **P6**, **P8** and **P9** degradation SEC traces.

Upon first inspection, the ^1^H NMR spectra for **P4** (Figure 4a) displays a large peak at δ = 4.07 ppm (*H^a^*), and historically this has been ascribed to the methylene protons adjacent to the ester (C(O)OC***H_2_***H_2_) within poly(MDO-*co*-styrene). Indeed, this is exactly where the peak appears within polyMDO **P5**, when ring-opened MDO is present between two MDO units (Figure 2a). However, an incorporation of 23 mol % ester links within polystyrene should furnish degraded oligomers of *M*_n_^theo^ = 500 Da, and this is significantly lower than the sample obtained (*M*_n_^oligo^ =1900 Da, *M*_w_^oligo^ = 9200 Da). As the conversion of each monomer aligns well with sample composition and the mass of copolymer obtained (94% yield), it is very likely that copolymer purification via precipitation does not cause any undesirable sample fractionation. Since, the original work published in the early 1980s, the kinetics of CKA polymerizations are much better understood [17], and CKA monomers are known to have very low reactivities, which can result in monomer composition drift under FRP conditions. We decided to repeat the polymerization of **P4** under identical conditions, reducing the time of polymerization to 6 h (**P6**). After purification, **P6** displayed a very similar molecular weight to **P4**, and crucially the peak at δ = 4.07 ppm (*H^a^*) was not visible in the NMR spectra of **P6** (Figure 4b). Interestingly, a small peak was present at δ = 3.71 ppm (*H^b^*), and we believe this peak corresponds to the methylene protons adjacent to the ester (C(O)OC***H_2_***H_2_) within the ring-opened MDO-centered triad with styrene on either side (Figure 2a). This small peak (*H^b^*) is also visible in the NMR spectra of **P4** (Figure 4a), and it is not surprising that this small peak has been overlooked in the past. The content of MDO, based on the 3.71 ppm peak, in **P6** was approximately 2 mol %, which corresponds well with its degraded oligomer sample (*M*_n_^theo^ = 5200 Da and *M*_n_^oligo^ = 4600 Da). Of course, at these lower reaction times, less composition drift has occurred, and we do not have the homopolymer of MDO present. The degraded oligomers of **P4** (*Đ* = 4.84, *M*_p_ = 10,100 Da) and **P6** (*Đ* = 2.69, *M*_p_ = 11,300 Da) possess very similar *M*_p_ values, and this suggests that the degradable polystyrene analogue present in both samples does contain a similar quantity of MDO (*H^b^*). The disparity in their degraded oligomer dispersities, which is clearly visible by SEC (Figure 5a), suggests **P6** has a much more uniform chemical composition distribution within the initial polymer sample, a theory confirmed by the lack of *H^a^* in **P6**. After degradation of **P6**, ^1^H NMR analysis (Figure 4c) displayed a small peak at δ = 3.51 ppm (*H^c^*), corresponding to the methylene protons adjacent to the terminal hydroxyl group (HOC***H_2_***H_2_) after ester hydrolysis (Figure 2a). The lack of *H^a^* when the reaction is performed at 6 h strongly suggests that the assignment of *H^b^* is accurate, and likewise the shift from δ = 3.71 ppm (*H^b^*) to δ = 3.51 ppm (*H^c^*) upon ester hydrolysis indirectly confirms the characterization of both *H^a^* and *H^b^*.

These initial results confirm that with extended polymerization durations, the formation of two distinct species can occur, with two very different chemical composition distributions. Over a 36 h polymerization (**P4**), the styrene monomers are predominantly consumed within the first 6 h, furnishing degradable polystyrene analogues with approximately 2 mol % MDO incorporated, followed by consumption of the remaining MDO units over the following 30 h, furnishing polyMDO with any residual styrene units incorporated. In the former case, MDO is primarily present between two styrene units (*H^b^*), whereas, in the latter case, MDO is primarily present between two MDO units (*H^a^*). Over a 6 h polymerization (**P6**), the styrene monomers are consumed with approximately 2 mol % MDO incorporation (*H^b^*) and, upon quenching, a sample is obtained without the presence of MDO-rich polymers (*H^a^*).

In order to confirm this hypothesis, BMDO and styrene were also copolymerized (**P10**) under equimolar conditions (120 °C, 36 h, 1 mol % DTBP, in bulk), and a purified sample with a BMDO composition of 22 mol % was obtained, comparable to the composition reported (31.1 mol %) by Bailey in 1982, as they used 2.5 mol % DTBP [4]. The ^1^H NMR spectra of **P10** (Figure 4d) displayed a large peak at δ = 5.12 ppm, which corresponds to the methylene protons (*H^d^*) adjacent to the ester (C(O)OC***H_2_***C) for the BMDO-centered triad and BMDO on either side (Figure 2b). A smaller peak is also visible at δ = 4.68 ppm, which corresponds to the methylene protons (*H^e^*) adjacent to the ester (C(O)OC***H_2_***C) for the BMDO-centered triad and styrene on either side (Figure 2b). A second copolymerization between BMDO and styrene was performed for 6 h (**P11**). As expected, the ^1^H NMR spectra of **P11** (Figure 4e) only displays *H^e^*, corresponding to approximately 2 mol % BMDO incorporation. These results confirm that monomer composition drift can be reduced, by reducing polymerization duration to the point at which styrene is near complete conversion. This is further confirmed by SEC analysis—**P10** (Figure 5b) displays a bimodal peak with a large dispersity (*Đ* = 3.83). After degradation, the oligomers obtained do not correlate well with the theoretical values (*M*_n_^theo^ = 600 Da, *M*_n_^oligo^ = 1900 Da and *M*_w_^oligo^ = 11,500 Da) and possess a very large dispersity (*Đ* = 6.05). Conversely, **P11** (Figure 5b) displayed a uniform peak with a lower dispersity (*Đ* = 2.10). After degradation, the oligomers obtained correlate well with the theoretical values (*M*_n_^theo^ = 5300 Da, *M*_n_^oligo^ = 5600 Da and *M*_w_^oligo^ = 14,000 Da) and possess a lower dispersity (*Đ* = 2.50). After degradation, the oligomers of **P10** and **P11** both displayed similar *M*_p_ values, *M*_p_^oligo^ = 16,200 Da and *M*_p_^oligo^ = 14,000 Da respectively, indicating that the degradable polystyrene analogue present in both initial samples contains a similar degree of BMDO incorporation (*H^e^*). After degradation of **P11**, ^1^H NMR analysis (Figure 4f) displayed a small peak at δ = 4.43 ppm (*H^f^*), corresponding to the methylene protons adjacent to the terminal hydroxyl group (HOC***H_2_***C) after ester hydrolysis (Figure 2b).

In order to increase the molecular weight of the degradable polystyrene analogues, we performed two copolymerizations with a reduced initiator concentration. An equimolar copolymerization of MDO and styrene was performed with 0.1 mol % DTBP, at 120 °C in bulk (**P7**)—under these conditions, 16 h is required to complete the consumption of styrene. **P7** possessed an increased molecular weight compared to **P6**, while the MDO incorporation and degraded oligomers were near identical (Table 1). This is expected, as the initiator concentration does not influence monomer reactivity ratios. Likewise, an equimolar copolymerization of BMDO and styrene was performed (**P12**) with 0.1 mol % DTBP, at 120 °C in bulk for 16 h. **P12** possessed an increased molecular weight compared to **P11**, while the BMDO incorporation and degraded oligomers were near identical (Table 1).

**Figure 6:**
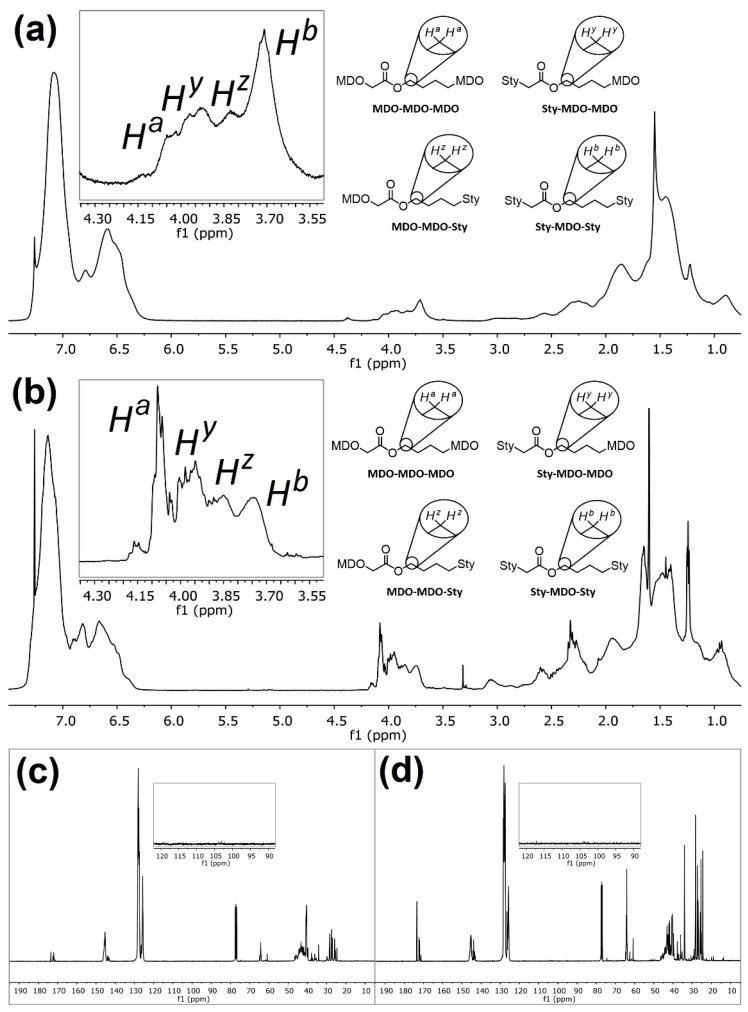
The ^1^H NMR (CDCl_3_) spectra of degradable poly(MDO-*co*-styrene) copolymers, specifically (**a**) **P8** (comprised of 12 mol % MDO) and (**b**) **P9** (comprised of 26 mol % MDO) included expansions of δ 4.30–3.55 ppm region displaying MDO methylene proton triad fractions adjacent to a main-chain ester (C(O)OC***H_2_***H_2_). The ^13^C NMR (CDCl_3_) spectra of degradable poly(MDO-*co*-styrene) copolymers, specifically (**c**) **P8** and (**d**) **P9**, including expansion of δ 90–120 ppm region confirming no presence of ring-retention MDO units.

With the aim of increasing MDO incorporation and consequently reducing degraded oligomer molecular weight, two copolymerizations were performed with varying monomer feed compositions under conditions which avoid the formation of MDO-rich polymers. Firstly, a ratio of 80:20 (MDO:styrene) was employed (1 mol % DTBP, 120 °C, for 6 h in bulk) and the resulting poly(MDO-*co*-styrene) copolymer (**P8**) was comprised of 88 mol % styrene and 12 mol % MDO. SEC analysis (Figure 5c) confirmed that the increased content of MDO in the feed lowered polymer molecular weight (*M*_n_ = 13,800 Da), as previously observed (Figure 3a). As intended, the degraded oligomer molecular weight (*M*_n_^oligo^ = 1,100 Da) was much lower than **P6** and **P7** (both 2 mol % MDO incorporation) and correlated nicely with the theoretical value calculated from the NMR MDO content (*M*_n_^theo^ = 900 Da) (Figure 5d). The ^1^H NMR spectra of **P8** (Figure 6a) was more complex than previous samples. Given the higher incorporation of MDO (12 mol %), multiple proton environments were observed for the methylene protons adjacent to the ester (C(O)OC***H_2_***H_2_) within poly(MDO-*co*-styrene). The proposed triad fractions, four in total, are illustrated in Figure 6a (inset). In addition to the previously described triad fractions of MDO between two MDO units (*H^a^*, MDO-MDO-MDO) and MDO between two styrene units (*H^b^*, Sty-MDO-Sty), we also observed two additional triad fraction of Sty-MDO-MDO (*H^y^*) and MDO-MDO-Sty (*H^z^*). The ^13^C NMR spectroscopy (Figure 6c) did not display a peak corresponding to the tertiary ketal carbon present in MDO ring-retained monomer units (typically approximately 100 ppm), removing any doubt that the four ^1^H NMR peaks (4.30–3.55 ppm) are a result of ring retention. Secondly, a ratio of 90:10 (MDO:styrene) was employed (1 mol % DTBP, 120 °C, for 6 h in bulk), and the resulting poly(MDO-*co*-styrene) copolymer (**P9**) was comprised of 74 mol % styrene and 26 mol % MDO. SEC analysis (Figure 5c) confirmed an even lower copolymer molecular weight (*M*_n_ = 9800 Da) and very low-molecular-weight degraded oligomers (*M*_n_^oligo^ = 600 Da) (Figure 5d), which also correlated well with the theoretical value calculated (*M*_n_^theo^ = 400 Da). The ^1^H NMR spectra of **P9** (Figure 6b) also displayed the four MDO ester methylene proton (C(O)OC***H_2_***H_2_) environments, in a similar fashion to **P8**. As the MDO incorporation (MDO 26 mol %) is even higher, the triad fractions are present in different proportions in comparison to **P8**, with MDO-MDO-MDO (*H^a^*) more prominent than Sty-MDO-Sty (*H^b^*). The ^13^C NMR spectroscopy (Figure 6d) did not display a peak corresponding to MDO ring retention.

## 4. Conclusions

Degradable polystyrene analogues have been synthesized via radical ring-opening copolymerization with two cyclic ketene acetal monomers, namely 2-methylene-1,3-dioxepane (MDO) and 5,6-benzo-2-methylene-1,3-dioxepane (BMDO), thereby incorporating ester bonds periodically within the main-chain polymer. Detailed NMR and SEC characterization of the resulting copolymers and their corresponding degraded oligomers has confirmed that significant monomer composition drift occurs at extended reaction times/higher conversions and high initiator concentrations, affording two distinct chemical composition distributions. After optimization of the reaction conditions, we are able to determine accurate degrees of ester incorporation and control molecular weight, and have revised the previously published ^1^H NMR spectra interpretations through more detailed assignments of triad sequences of poly(styrene-*co*-MDO) and poly(styrene-*co*-BMDO). As such, we have resolved the previous important discrepancies in the literature. Our future work in this space will focus on a comprehensive characterization of the physical, thermal and mechanical properties of these materials.

## Figures and Tables

**Table 1:** Degradable polystyrene analogues via copolymerization with CKAs, and degraded styrenic oligomers after main-chain ester hydrolysis. All polymerizations are performed in bulk at 120 °C, with 50 mmol total monomer. *^a^* Determined by ^1^H NMR (CDCl_3_) spectroscopy. *^b^* Determined by SEC. *M*_n_^theo^ = theoretical number average molecular weight of degraded oligomers, MDO = 2-methylene-1,3-dioxepane, BMDO = 5,6-benzo-2-methylene-1,3-dioxepane, DTBP = di-*tert*-butyl peroxide, and *Đ* = *M*_w_/*M*_n_.

Entry	CKA	Sty:CKA:DTPB	Time (h)	Monomer Conversion *^a^*		Sample Composition *^a^*		Polymer Characterization *^b^*			Degraded Oligomer Characterization *^b^*			
				Sty (%)	CKA (%)	Sty	CKA	*M*_n_ (Da)	*M*_w_ (Da)	*Đ*	*M*_n_^theo^ (Da)	*M*_n_^oligo^ (Da)	*M*_w_^oligo^ (Da)	*Đ*
**P1**	-	100:0:1	36	99	-	1	0	17,800	93,400	5.25	-	-	-	-
**P2**	MDO	80:20:1	36	99	20	0.95	0.05	35,100	91,700	2.61	2100	4800	26,900	5.60
**P3**	MDO	65:35:1	36	99	26	0.88	0.12	32,600	79,800	2.45	900	3000	16,100	5.37
**P4**	MDO	50:50:1	36	99	30	0.77	0.23	29,200	79,500	2.72	500	1900	9200	4.84
**P5**	MDO	0:100:1	36	-	78	0	1	20,600	41,200	2.00	-	-	-	-
**P6**	MDO	50:50:1	6	93	2	0.98	0.02	35,600	80,200	2.25	5200	4600	12,400	2.69
**P7**	MDO	50:50:0.1	16	90	2	0.99	0.02	80,300	173,500	2.16	5200	4300	12,200	2.83
**P8**	MDO	20:80:1	6	96	3	0.88	0.12	13,800	28,200	2.04	900	1100	2,700	2.45
**P9**	MDO	10:90:1	6	97	4	0.74	0.26	9800	17,300	1.77	400	600	1300	2.17
**P10**	BMDO	50:50:1	36	99	22	0.82	0.18	17,400	66,600	3.83	600	1900	11,500	6.05
**P11**	BMDO	50:50:1	6	96	2	0.98	0.02	42,500	89,100	2.10	5300	5600	14,000	2.50
**P12**	BMDO	50:50:0.1	16	98	2	0.98	0.02	50,100	126,600	2.53	5300	4600	13,500	2.93
